# Effectiveness comparison of a Chinese dicitraditionalmene formula Wuzi Yanzong Pill and its analogous prescriptions for the treatment of oligoasthenozoospermia: A systematic review and meta-analysis protocol

**DOI:** 10.1097/MD.0000000000015594

**Published:** 2019-05-17

**Authors:** Yongqiang Li, Yahui Xue, Binghao Bao, Jisheng Wang, Hengheng Dai, Xiaoyong Gong, Wei Zheng, Yanfeng Li, Bao Zhang

**Affiliations:** a Department of Andrology, The Second Affiliated Hospital of Shaanxi University of Traditional Chinese Medicine, Shaanxi; b Department of Andrology; c Department of Urology, Dongzhimen Hospital, Beijing University of Chinese Medicine, Beijing, China.

**Keywords:** oligoasthenozoospermia, protocol, systematic review, traditional Chinese medicine, Wuzi Yanzong Pill

## Abstract

**Background:**

Among male sterility factors, oligoasthenozoospermia is the most common. As people's lifestyle changes and the population ages, the incidence of oligoasthenozoospermia continues to increase. The studies have shown that about 15% of married couples in the world are affected by infertility, among which infertility caused by male factors alone accounts for about 50%. Many clinical trials have proven that Wuzi Yanzong Pill has a significant effect in the treatment of oligoasthenozoospermia. In this systematic review, we aim to evaluate the effectiveness and safety of Wuzi Yanzong Pill for oligoasthenozoospermia.

**Methods:**

We will search for PubMed, Cochrane Library, AMED, EMbase, WorldSciNet; Nature, Science online, and China Journal Full-text Database (CNKI), China Biomedical Literature CD-ROM Database (CBM), and related randomized controlled trials included in the China Resources Database. The time is limited from the construction of the library to April 2019. We will use the criteria provided by Cochrane 5.1.0 for quality assessment and risk assessment of the included studies, and use the Revman 5.3 and Stata13.0 software for meta-analysis of the effectiveness, recurrence rate, and symptom scores of oligoasthenozoospermia.

**Ethics and dissemination:**

This systematic review will evaluate the efficacy and safety of Wuzi Yanzong Pill for treating oligoasthenozoospermia. Because all of the data used in this systematic review and meta-analysis has been published, this review does not require ethical approval. Furthermore, all data will be analyzed anonymously during the review process Trial.

**Trial registration number:**

PROSPERO CRD42019119170

## 1 Introduction

According to the World Health Organization Human Semen Examination and Handling Laboratory Manual,^[1,2]^ oligoasthenozoospermia refers to the detection of male semen using modern medical computer-aided analysis (CASA), if the number of sperm per milliliter of semen is <20 × 10^6^ it is called oligospermatism; if the sperm in the semen parameter index is <50% of the sperm (a and b) or the sperm of the a-class movement is <25%, it is called asperospermia.^[3]^ Clinically, oligozoospermia often coexists with asthenospermia, so it is called oligoasthenozoospermia. Currently, about 15% of married couples in the world are affected by infertility, among which infertility caused by male factors alone accounts for about 50%.^[4,5]^ Among male sterility factors, oligoasthenozoospermia is the most common. The important reason, but its cause is complicated.^[6,7]^ The common causes of oligoasthenozoospermia have endocrine factors, reproductive tract infections, chromosomal abnormalities, cryptorchidism, varicocele and systemic diseases, as well as genetic, metabolic, and immune dysfunction. Seasonal changes, long-term wetlands, mental stimulation or trauma, surgery, etc. may induce or aggravate the disease.^[8]^ Because of its stubborn and difficult recurrence rate, the cure for oligoasthenozoospermia has long been a major problem in the world of medicine.

For the treatment of oligoasthenozoospermia, modern medicine mainly treats the primary cause and the application of various hormones.^[9]^ The main treatments include general treatment, endocrine therapy, anti-infective therapy, surgical treatment, and assisted reproductive technology (ART).^[10,11]^ Hormone medications mainly include gonadotropin-releasing hormone (GnRH), low-dose androgen, estrogen receptor antagonists, and growth hormone. Because oligoasthenozoospermia is not an independent disease, it is the result of multiple diseases or factors.^[12,13]^ This makes the treatment of western medicine have certain limitations, and the clinical efficacy is not ideal. Gene therapy is currently in the research stage and has not been promoted in clinical practice.^[14]^ With the development of medicine, assisted reproductive technology as a new treatment method has effectively improved the conception rate of patients with oligoasthenozoospermia, but it has not fundamentally improved the sperm quality of patients.

Oligoasthenozoospermia belongs to the category of “infertility,” “no child,” and “sperm cold” in Traditional Chinese medicine (TCM).^[15,16]^ TCM believes that sperm production and maturity are closely related to kidney deficiency. Kidney is the innate foundation and is the key link in the development and reproduction of human life. Therefore, TCM believes that kidney deficiency is the main pathogenesis of semen abnormalities.^[17]^ Based on this, the unique methods of syndrome differentiation, classification, and individualized treatment of TCM have pointed out the direction for the treatment of oligoasthenozoospermia and get satisfactory results. In the long-term clinical practice, we found that Chinese medicine used the highest frequency of Wuzi Yanzong Pill in the treatment of this disease.^[18]^ Wuzi Yanzong Pill originated from the famous traditional Chinese medicine prescriptions in the Tang Dynasty and have been widely used for improving semen quality and treating infertility for a long time. Wuzi Yanzong Pill has a long history of treating this disease, its clinical effect is remarkable, and there are relatively many clinical reports.^[19,20]^ However, the experimental design and quality of these studies are uneven, which affects the reliability of the research conclusion to some extent. This has made the studies results difficult to be recognized by the medical community. Therefore, we expect a meta-analysis to evaluate the efficacy and safety of Wuzi Yanzong Pill in the treatment of oligoasthenozoospermia in order to provide clues for clinical application and research.

## 2 Methods

This is a systematic review and ethical approval was not necessary.

### 2.1 Study registration

This systematic review protocol has been registered on PROSPERO as CRD42019119170. (http://www.crd.york.ac.uk/PROSPERO/display_record.php?ID=CRD42019119170).

### 2.2 Eligibility criteria

#### 2.2.1 Type of study

Take Wuzi Yanzong Pill or Wuzi Yanzong Pill combined with other effective interventions as main treatment, including randomized controlled trials of the control group (effective methods other than traditional Chinese medicine). Language is limited in Chinese and English. Non-randomized controlled trials, quasi-randomized controlled trials, case series, case reports, and crossover studies will be excluded.

#### 2.2.2 Participants

The patients conforming to the diagnostic criteria established in the Management Guidelines of Male Infertility issued by the Canadian Urological Association (CUA) in 2015, the Guidelines for Clinical Diagnosis and Treatment of Male Infertility in 2005, Chinese Medical Association, or other authoritative diagnostic criterias. And the patients must be older than 18 years old.

#### 2.2.3 Types of interventions

##### 2.2.3.1 Experimental interventions

The Chinese medicine preparation Wuzi Yanzong Pill or the combined western medicine are used as experimental interventions. Both prescription and Chinese patent medicines will be included. Other traditional Chinese medicine treatments such as intravenous medication, acupuncture, and massage will be limited.

##### 2.2.3.2 Control interventions

As for the control interventions, who accepted simple western medicine can be used as a control intervention or didn‘t get any treatment as a blank control would be adopted. However, once they had accepted the therapy of TCM, the trials will be rejected.

#### 2.2.4 Outcomes

##### 2.2.4.1 Primary outcomes

The primary outcome measurement will be male semen parameters. It will be routinely tested for semen according to WHO's semen analysis criteria. And the sperm concentration, sperm motility, sperm morphology will be included.

##### 2.2.4.2 Secondary outcomes

We also need to pay attention to the following outcomes: sperm volume, sperm DNA fragmentation, and activity of acrosomal enzyme. More importantly, the adverse reactions of patients during medication will also be taken seriously.

#### 2.2.5 Data source

##### 2.2.5.1 Electronic searches

Database Search: PubMed, Cochrane Library, AMED, EMbase, WorldSciNet, Nature, Science online and China Journal Full-text Database (CNKI), China Biomedicalstudies CD-ROM Database (CBM), China Resources Database. A studies review of clinical studies on Wuzi Yanzong Pill for the treatment of oligoasthenozoospermia published in domestic and foreign biomedical journals from the establishment of the library to April 2019. Based on the standards of the Cochrane Collaboration Workbook of the International Evidence-Based Medicine Center, a manual and computer-based approach is used to conduct relevant studies searches. Search terms include: Chinese medicine, traditional Chinese medicine, Wuzi Yanzong Pill/Bolu, oligoasthenozoospermia, oligospermatism, and asthenospermia. The complete PubMed search strategy is summarized in Table 1.

**Table 1:**
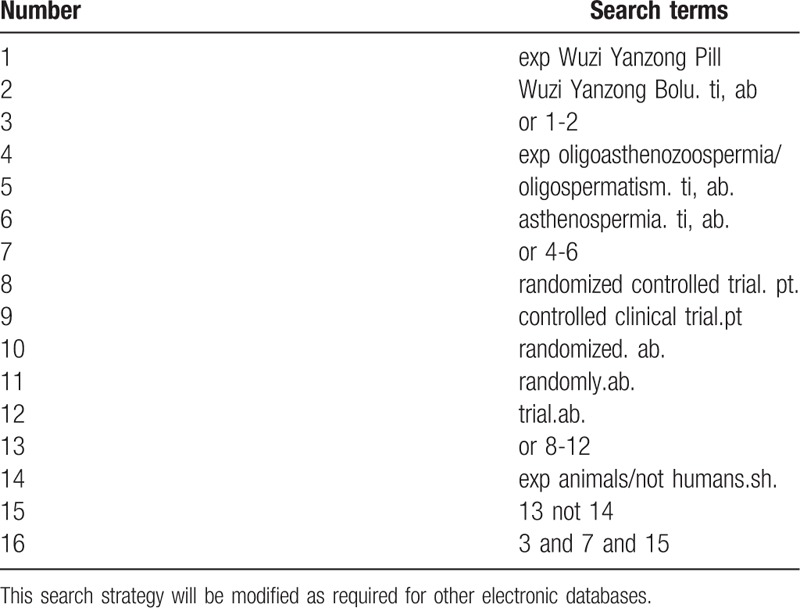
Search strategy used in PubMed database.

##### 2.2.5.2 Searching other resources

The manual search is mainly for dissertations, ongoing experiments, and grey literature. We will look for abstracts of dissertations, conference papers, and conference papers related to Wuzi Yanzong Pill and oligoasthenozoospermia. Ongoing trials for the new reviews that are relevant to this term will be retrieved from the WHO International Clinical Trials Registry Platform (ICTRP), ClinicalTrials.gov, and the Chinese Clinical Trial Registry. For ongoing experiments, we will try to contact the trial author to help provide up-to-date clinical data. Potential gray literature will be elected in OpenGrey.eu. website.

#### 2.2.6 Data collection and analysis

**Figure 1:**
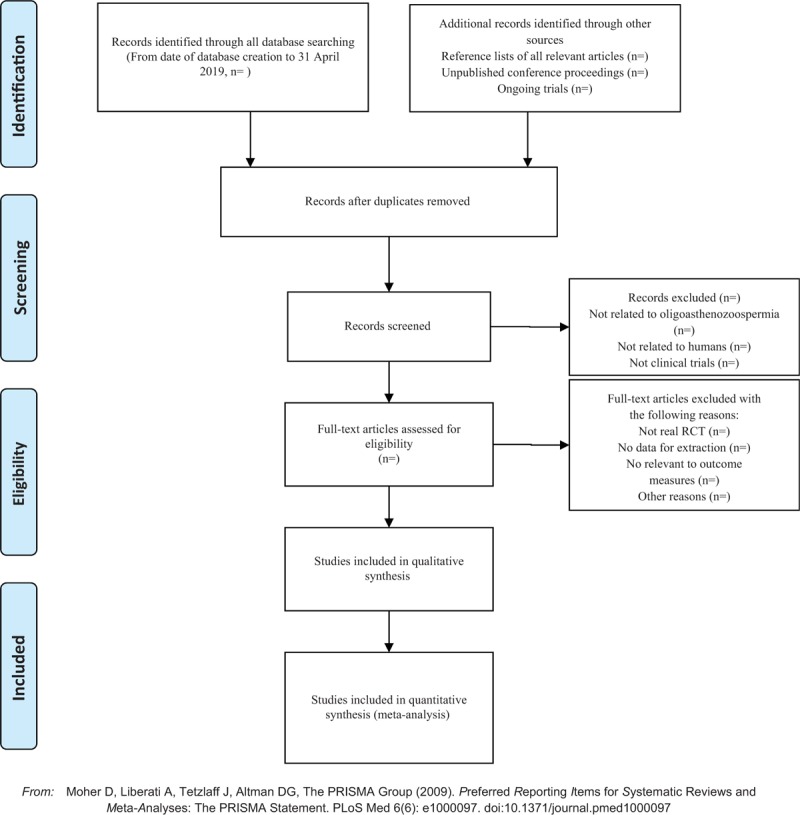
The PRISMA flow chart.

Applying the EndnoteX7 software to manage the included references. Two qualified evaluators independently screened the titles and abstracts of the selected studies, excluding duplicates and documents that did not significantly conform to the study. After a preliminary evaluation, the selected documents will be read one by one. Exclusions were based on inclusion criteria for uncontrolled studies, no randomization, inconsistent assessment criteria, and similar data. If there are different opinions, the third reviewer should be consulted. Studies information and data extraction were carried out on the final included studies, including the experimental methods of the study, the basic information of the included cases, the observation period, the intervention methods, observation indicators, and test results of the treatment group and the control group. The details of selection process will be shown in the PRISMA flow chart (Fig. 1).

#### 2.2.7 Risk of bias

The quality of the studies will be assessed by using the Cochrane Handbook 5.1.0 (Cochrane Handbook 5.1.0). The assessment will include random sequence generation, randomization correctness, allocation scheme hiding, blinding of patients and implementers, accuracy of data results, and other risk of bias. The risk of low bias is expressed as “low risk” and the risk of high bias is expressed as “high risk.” The information provided in the studies is inaccurate or does not provide sufficient information for the bias assessment to be expressed as “unclear risk.” The above content evaluation was independently evaluated by 2 researchers, and any differences will be resolved through discussions with the third reviewer.

#### 2.2.8 Statistical analysis

The meta-analysis in this study will use Rev Man5.3 and Stata13.0 statistical software. Heterogeneity tests will be used for the included experimental studies. The numerical variable will be expressed as the normalized mean difference (SMD) with a confidence interval (CI) of 95%. The heterogeneity of each pairwise comparison will be tested by chi-square test (test level *α* = 0.1). If there is no heterogeneity, a fixed effect model will be used. If there is significant heterogeneity between a set of studies, we will use a random effects model (REM) for meta-analysis. We will explore the reasons for the existence of heterogeneity from various aspects such as the characteristics of the subjects and the degree of variation of the interventions. The source of heterogeneity is further determined by means of sensitivity analysis.

#### 2.2.9 Publication bias

If a result of a meta-analysis contains >10 articles and above, we will use a funnel plot to test the risk of publication bias. Quantitative methods such as Begg testing and Egger testing will be used to help assess publication bias in the application.

#### 2.2.10 Quality of evidence

The GRADE method will be used to assess the quality of evidence for key outcomes. This assessment will be conducted through a Guideline Development Tool (GRADEpro GDT, https: //gradepro.org/).

## 3 Discussion

In recent years, with the changes in people's lifestyles and the aging of the population, the incidence of oligoasthenozoospermia has increased.^[21]^ Traditional Chinese medicine believes that the etiology and pathogenesis of oligoasthenozoospermia is related to spleen and kidney deficiency and qi and blood block. TCM can play the role of strengthening the spleen and kidney, promoting blood circulation and collaterals, and at the same time can improve the mood and achieve the purpose of treatment.^[22]^ Wuzi Yanzong Pill originated from the famous traditional Chinese medicine prescriptions in the Tang Dynasty and have been widely used for improving semen quality and treating infertility for a long time. Recent studies have shown that Wuzi Yanzong Pill can significantly improve cAMP content and expression of Catsper1 protein in testis tissue of rats with oligozoospermia, thereby improving sperm quality.^[23]^ With the deepening of understanding of oligoasthenozoospermia, the trials and clinical reports of Wuzi Yanzong Pill for oligoasthenozoospermia have gradually increased. Whether it is syndrome differentiation or special disease, Wuzi Yanzong Pill has achieved good results in the treatment of oligoasthenozoospermia. To the best of our knowledge, there has been no comparison of the efficacy and safety of Wuzi Yanzong Pill in the treatment of oligoasthenozoospermia in recent years. Therefore, we will compare the effectiveness and safety of Wuzi Yanzong Pill in the treatment of oligoasthenozoospermia with systematic evaluation and meta-analysis. The results of this study can provide a possible ranking for the treatment of oligoasthenozoospermia by Chinese medicine. We hope that the results will provide clinicians with the best options for treating oligoasthenozoospermia and provide research directions. Although we will conduct a comprehensive search in this study, languages other than Chinese and English will be restricted, which will lead to some bias. In addition, the relevant literature on the treatment of oligoasthenozoospermia in Chinese medicine is small and the overall quality is low, which may affect the authenticity of this study. Therefore, we hope that in the future, we will have a more rigorous and reasonable multicenter randomized controlled trial to explore the clinical efficacy of oligoasthenozoospermia in the treatment of oligoasthenozoospermia, so that the conclusion is more objective and reasonable.

## Author contributions

**Data curation:** Yahui Xue, Yongqiang Li, Binghao Bao.

**Formal analysis:** Yongqiang Li, Yahui Xue, Hengheng Dai, Jisheng Wang.

**Funding acquisition:** Yanfeng Li.

**Investigation:** Hengheng Dai, Xiaoyong Gong, Yanfeng Li.

**Methodology:** Wei Zheng.

**Project administration:** Wei Zheng, Yanfeng Li, Bao Zhang.

**Resources:** Yahui Xue, Binghao Bao.

**Software:** Yongqiang Li, Yahui Xue.

**Writing – original draft:** Yongqiang Li, Jisheng Wang, Hengheng Dai, Bao Zhang.

**Writing – review & editing:** Yanfeng Li, Bao Zhang.
